# Prevalence of Antimicrobial Resistant and Virulent *Salmonella* spp. in Treated Effluent and Receiving Aquatic Milieu of Wastewater Treatment Plants in Durban, South Africa

**DOI:** 10.3390/ijerph120809692

**Published:** 2015-08-18

**Authors:** Ejovwokoghene C. Odjadjare, Ademola O. Olaniran

**Affiliations:** Discipline of Microbiology, School of Life Sciences, College of Agriculture, Engineering and Science, University of KwaZulu-Natal, Westville Campus, Private Bag X54001, Durban 4000, South Africa; E-Mail: ecodjadjare@yahoo.com

**Keywords:** antimicrobial resistance, *Salmonella* spp., treated effluent, virulence genes, wastewater, physicochemical parameters

## Abstract

In this study, we evaluated the impact of treated wastewater effluent from two wastewater treatment plants on the physicochemical parameters and *Salmonella* spp. load of receiving rivers. Presumptive *Salmonella* spp. were obtained at all sampled points including the discharge points, with counts ranging from 0 to 4.14 log cfu/mL at both plants. Turbidity, chemical and biological oxygen demand were found to be high and mostly above the required limit for treated wastewater discharge. However, recorded nitrate and phosphate values were very low. Of the 200 confirmed *Salmonella* spp. isolates recovered from the treated effluent and receiving surface waters, 93% harbored the *spi*C gene, 84% harbored the *misL* gene, and 87.5% harbored the *orf*L gene while 87% harbored the *pip*D gene. The antibiotic resistance profile revealed that the isolates were resistant to sulfamethoxazole, nalidixic acid and streptomycin, but susceptible to quinolones and third generation β-lactams. These results indicate that in South Africa treated effluents are still a major source of contamination of rivers with pathogens such as *Salmonella*. Appropriate steps by the regulatory authorities and workers at the treatment plants are needed to enforce stipulated guidelines in order to prevent pollution of surface water resources due to the discharge of poorly treated effluents.

## 1. Introduction

South Africa is a water stressed country due to the low average received rainfall (465 mm), which is below the global average of 860 mm [1]. Demand for this important scarce resource is expected to increase due to rapid industrial development, increasing human population, *per capita* consumption increases and the resulting impact of human activities on the environment [2,3]. High water demand and consumption also leads to increases in the volume of wastewater generated [4]. The availability of good quality water is of paramount importance bringing to the fore the consequence of contamination of water bodies with pathogenic microorganisms [5]. Water bodies such as rivers are subject to dramatic changes in microbial and physicochemical qualities as a result of a variety of anthropogenic activities in the watershed. These changes are caused by discharges of municipal raw waters or treated effluent at a specific point source into the receiving surface waters [6,7]. Point-source pollution problems will not only increase treatment costs considerably, but may also introduce a wide range of pathogens and harmful chemicals into surface waters that may be supplied to many rural and urban communities, thus resulting in incidences of waterborne diseases with far reaching socio-economic implications [8,9]. Although a vast majority of microorganisms present in wastewater are not pathogenic [10], some pathogenic bacteria possibly originating from discharge of inadequately treated wastewater effluent have been implicated in the outbreak of waterborne diseases over the years [11].

*Salmonella* spp. are important Gram-negative bacilli, which infect both humans and animals causing a wide range of diseases such as gastroenteritis, typhoid fever, osteomyelitis, septicemia and meningitis. This genus comprises of over 2000 recognized serotypes and is divided into two species namely *S. bongori* and *S. enterica*. *Salmonella enterica* consist of six subspecies, namely *enterica, arizonae, salamae, diarizonae, houtenae and indica* [12,13]. It is estimated that 93.8 million cases of gastroenteritis due to *Salmonella* spp. occur globally each year, with some 155,000 deaths [14]. This high number of infections emphasizes the importance of this intracellular pathogen and indicates a considerable worldwide disease burden. Mortality rate of *Salmonella* infections is a health problem mainly in developing countries [15], while morbidity due to acute *Salmonella* infection can also have socio-economic impacts in developed countries [16]. Added to this disease burden are the complications arising from the inefficacy and failures of antimicrobial chemotherapies applied in clinical practice to remedy these diseases. Bacterial resistance to antibiotics has increased globally in recent years and poses threats to human health [17]. Antimicrobial resistance in *Salmonella* has been associated with an increase in the number of adverse events following infection such as higher levels of hospitalization, longer illness, and higher risk of invasive illness as well as treatment failures [18]. The World Health Organization (WHO), the European Commission and the United States Centre for Disease Control and prevention (CDC) have recognized the importance of studying the emergence of resistance genes as well as the need for control strategies [19]. In most countries, the microbial quality of final treated effluent is estimated based on the level of indicator organisms present [20,21]. However, several studies have shown that the presence of indicator organisms does not always correlate with the presence of pathogens [22,23]. This study therefore aimed to determine the prevalence of antibiotic resistant and virulent *Salmonella* spp. in treated effluents and receiving surface water of two wastewater treatment plants located in Durban, South Africa.

## 2. Materials and Methods

### 2.1. Description of Sampling Site and Sample Collection

Two wastewater treatment plants, namely the Northern Wastewater Treatment Works (NWWTW) and the New Germany Wastewater Treatment Plant (NGWTP) located in Durban, South Africa were sampled and studied. Details of these treatment plants can be found elsewhere [24]. The NWWTW and NGWTP discharge their final effluents into the Umgeni and Aller rivers, respectively. Water samples were collected monthly from both wastewater treatment plants at the clarifier before chlorination (B.C), discharge point after chlorination (D.P), 500 meters upstream (U.S) and 500 meters downstream (D.S) of the discharge point between March 2012 and February 2013. Samples were collected in 5 L plastic containers disinfected 24 h prior to collection by rinsing with deionized water and soaking in 70% ethanol. During collection of samples, the containers were rinsed with the sampled water before filling (at a depth of approximately one metre at each sampling point) to three-quarter of the container leaving space to allow for proper mixing. The collected samples were placed in ice packs, transported to the laboratory at the Department of Microbiology, University of KwaZulu-Natal (Westville) and processed within 24 h of collection.

### 2.2. Physico-Chemical Analysis of Water Samples

Temperature of the water samples was measured on site with a mercury thermometer; the pH was determined using a pH meter (Beckman, CA, USA) while turbidity was measured with a turbidimeter (21000P, HACH, CO, USA). Biochemical oxygen demand (BOD_5_) was determined using the LDC 101 probe with an HQ40d multimeter (HACH) after incubation for a period of 5 days according to the manufacturer’s instructions. Chemical oxygen demand (COD) was measured with a Nova 60 spectroquant (Merck, Darmstadt, Germany) according to the manufacturer’s instructions. Conductivity and total dissolved solids (TDS) were measured using the CDC 401 probe and HQ40d multimeter (HACH Colorado, USA). Nitrate (NO_3_) and Phosphate (PO_4_) concentrations were determined at Aquatico Laboratories (Pretoria, South Africa) using 50 mL of water samples.

### 2.3. Microbial Profile of Treated Wastewater Effluent and Receiving River

In order to avoid overestimation, *Salmonella* present in the water samples was quantified via a standard membrane filtration technique as previously described [3]. Ten-fold serial dilutions of the water samples were made and membrane filter (Pall Corporation, MI, USA) of 0.45 µm pore size and 47 mm diameter was used to concentrate 50 mL of appropriately diluted water sample. The membrane filter was then placed on the surface of *Salmonella-Shigella* (SS) agar and incubated aerobically at 37 °C for 18 to 24 h to enumerate *Salmonella* spp. Colourless colonies with or without black center depending on the production of hydrogen sulphide were enumerated as presumptive *Salmonella* spp. [25]. The organisms are referred to as presumptive *Salmonella* because certain bacteria also exhibit the same characteristics as *Salmonella* on SS agar.

### 2.4. Enrichment and Isolation of Salmonella spp. from Water Samples

Isolation of *Salmonella* spp. from the water samples was done by a previously described enrichment method [26] with modifications. Thoroughly mixed water samples (25 mL) were added to 250 mL of sterile buffered peptone water and incubated at 37 °C for 18 to 24 h with shaking at 230 rpm. Thereafter, 1 mL of the pre-enrichment solution was appropriately diluted in 9 mL of sterile Rappaport-Vassiliadis soy broth (RVS) (Oxoid, Basingstoke, UK) depending on the turbidity of the water sample used in the pre-enrichment and incubated at 42 °C for 24 to 48 h with shaking at 230 rpm. One hundred microliters (100 µL) of the appropriately diluted RVS broth was spread-plated on *Salmonella* chromogenic agar (Oxoid) in duplicate and incubated aerobically at 37 °C for 18 to 24 h. Colonies with purple color were isolated and purified on fresh nutrient agar plates and subjected to further identification using biochemical tests and molecular method.

#### 2.4.1. Biochemical Screening of Presumptive *Salmonella* spp.

Triple sugar iron (TSI), Simmons citrate, lysine iron agar (LIA) and urea agar (Oxoid) slants were prepared according to the manufacturer’s instructions and inoculated with a 24 h nutrient agar-grown culture of the isolates incubated at 37 °C. The surface of the agar slant was inoculated using a sterile inoculating loop while a stab was made at the center of the slant using a sterile inoculating needle. The tubes were then incubated under aerobic conditions at 37 °C for 24 to 48 h. Tubes exhibiting alkaline slant and acidic butt with H_2_S production on TSI slants, purple color in butt of LIA tube, blue color development on slant of citrate agar and no color change on urea indicated positive results for *Salmonella* spp. Isolates showing biochemical reaction consistent with *Salmonella* spp. were further confirmed using molecular based method.

#### 2.4.2. Molecular Confirmation of Presumptive *Salmonella* spp.

Template DNA was prepared from 24 h freshly grown cultures of the isolates on nutrient agar using the boiling method as previously described [27] with modifications. Well isolated colonies (3 to 5) were suspended in 70 µL of sterile deionized water, boiled in a water bath at 100 °C for 10 min and cooled on ice for a further 5 min. Thereafter, the suspension was centrifuged at 13,000 rpm in a micro-centrifuge (Eppendorf 5415D, Hamburg, Germany) for 5 min. The supernatant (50 µl) was carefully transferred to a sterile eppendorf tube and used as a template in the PCR assay. *Salmonella* spp. was confirmed by the amplification of the *inv*A gene using the primers F-5′-TGC-CTA-CAA-GCA-TGA-AAT-GG-3′ and R-5′-AAA-CTG-GAC-CAC-GGT-TGA-CAA-3′ [28]. The PCR mixture contained: 1 × PCR reaction buffer, 1 mM of MgCl_2_, 200 µM of each dNTPs, 0.5 µM of each primer, 2 U of *Taq* polymerase (Supertherm, Inqaba, Pretoria, South Africa) and 2 µL of template DNA in a final volume of 25 μL. Amplification was performed in a thermocycler (Bio-Rad T100, Singapore) with a temperature regime of 2 min at 94 °C for initial denaturation followed by 35 cycles of 94 °C for 1 min, annealing at 58 °C for 1 min, extension at 72 °C for 1 min with a final extension step at 72 °C for 5 min. PCR mixture with 2 µL of molecular grade water was used as negative control while *S. typhimurium* ATCC 13317 was used as a positive control. The PCR products were examined by electrophoresis in a 1.5% (w/v) agarose gel at 60 V for 90 min in 1% TAE buffer and visualized via UV illumination (Syngene Cambridge, UK) after staining in 1 mg/mL ethidium bromide for 15 min.

#### 2.4.3. Virulence Gene Detection

The isolates were evaluated for the presence of virulence genes in *Salmonella* pathogenicity island (SPI) 2 through 5 using the primers shown in Table 1 [29,30]. The presence of *mis*L and *orf*L virulence genes was confirmed using duplex PCR method developed in this study, while *spi*C and *pip*D were detected in separate single PCR assay. The reaction was done in a 25 μL reaction volume consisting of 1 × buffer, 1 mM MgCl_2_, 0.2 mM dNTPs, 0.5 μM of each primer, 2 μL of template DNA and 2.5 U of *Taq* polymerase (Supertherm). Amplification was carried out in a thermocycler (Bio-Rad T100) using a temperature program consisting of initial denaturation of 94 °C for 2 min, followed by 35 cycles of 94 °C for 1 min, 1 min at the respective annealing temperature of various primers (Table 1), 72 °C for 1 min with a final extension at 72 °C for 5 min. PCR mixture with 2 µL of molecular grade water was used as negative control while *S. typhimurium* ATCC 13317 was used as a positive control. The amplicons were examined by electrophoresis in a 1.5% agarose gel at 60 V for 90 min, stained in 1 mg/mL ethidium bromide for 5 min and viewed under UV light (Syngene Cambridge, UK).

#### 2.4.4. Antibiotic Resistance Determination

Antibiotic susceptibility of the isolates was determined using the Kirby-Bauer disk diffusion method. The isolates were screened against a predetermined and commercially available panel of 20 antibiotics (Oxoid), belonging to six classes. Fresh culture were grown overnight in Mueller-Hinton broth and standardized to 0.5 McFarland by diluting with sterile Mueller-Hinton broth until an optical density of 0.08 to 0.1 was obtained on a spectrophotometer (Shimadzu UV-1800, Kyoto, Japan) at 625 nm. The standardized culture of the isolates were inoculated onto Mueller-Hinton agar using sterile swabs for confluence growth and allowed to dry for 10 min. Thereafter, appropriate antibiotic disks were placed at equidistance on the surface of the agar plates with a sterile forceps and incubated at 37 °C for 24 h. The diameter of the zone of inhibition was measured to the nearest millimeter and interpreted using charts recommended by the CLSI [31].

### 2.5. Statistical Analysis

Mean values of results and standard deviation were calculated using Microsoft Excel 2010 edition. Pearson’s correlation was determined using the SPSS 21.0 for windows program (SPSS, Inc. WA, USA) after normalization of skewed datasets. Correlations were considered statistically significant at *p* values of <0.05.

**Table 1:** Primer used for the detection of virulence genes in *Salmonella* spp. recovered from treated wastewater effluent and receiving surface waters.

Gene Target	Oligonucleotide Sequence (5ʹ-3ʹ)	Amplicon Size (bp)	Annealing Temperatures (°C)
*spi*C	f-CCTGGATAATGACTATTGAT	309	54
	r-AGTTTATGGTGATTGCGTAT		
*pip*D	f-CGGCGATTCATGACTTTGAT	400	56
	r-CGTTATCATTCGGATCGTAA		
*mis*L	f-GTCGGCGAATGCCGCGAATA	550	60
	r-GCGCTGTTAACGCTAATAGT		
*orf*L	f-GGAGTATCGATAAAGATGTT	350	60
	r-CGTTATCATTCGGATCGTAA		

## 3. Results

### 3.1. Physicochemical Profile of Water Samples

The physicochemical parameters of treated effluent at the NWWTP and NGWTP and the receiving surface waters are shown in Table 2 and Table 3. Temperature was stable across all sampled points in each month but seasonal variations were observed. The lowest temperature recorded was 12 °C, while the highest temperature of 27 °C. The pH was stable at all sampled points in each month but varied throughout the duration of the study. It ranged from 6.41 (at the U.S in September) to 7.88 (at the D.P in February) at the NWWTW while at the NGWTP, the pH ranged from 6.30 (at the U.S in July) to 8.00 (at the D.S in February). Turbidity values recorded varied across all sampled point and month with no significant decrease in turbidity obtained at the D.P compared to B.C point (Table 2). At the NGWTP, recorded values ranged from 1.42 NTU at D.P in April to 40.40 NTU at U.S in August. The COD values varied highly throughout the study period ranging from 22.33 to 313 mg/L at U.S at the NGWTP. Some levels of decrease at the D.P was observed only in the months of April (36.5%), May (21.11%) and June (55.6%) but increased in the other months. Recorded COD values also varied highly at the NWWTW and ranged from <10 mg/L in March and May (at the D.P) to 312.44 mg/L in July (at the D.S). Some level of decrease in COD was recorded at the D.P in May, August, October and December but increased in other months especially in March and May from <10 mg/L to 309 mg/L. Stable BOD_5_ values were observed across each sampled point in each month ranging from 1.03 mg/L to 9.42 mg/L at the NWWTW. The values of BOD_5_ at the D.S were higher than values recorded at other sampled points and ranged from 3.58 mg/L to 7.74 mg/L. At the NGWTP, the BOD_5_ was stable across all sampled points in each month but varied throughout the study period ranging from 2.20 mg/L to 11.04 mg/L.

**Table 2:** Physicochemical parameters of wastewater effluent from Northern wastewater treatment works and the receiving river from March 2012 to February 2013.

MONTH	Sampled Point	Parameter (Mean ± SD)						
		T (°C)	pH	T (ntu)	COD (mg/L)	BOD (mg/L)	NO_3_ (mg/L)	PO_4_ (mg/L)
	U.S	26.00 ± 0.00	7.25 ± 0.09	16.67 ± 0.38	161.33 ± 4.37	5.62 ± 1.01	1.06	0.05
MAR	B.C	26.00 ± 0.00	7.11 ± 0.05	7.91 ± 0.33	104.78 ± 13.73	2.23 ± 0.36	0.06	3.51
	D.P	25.00 ± 0.00	7.36 ± 0.07	23.40 ± 12.13	<10 ± 0.00	5.13 ± 0.18	0.06	3.47
	D.S	26.00 ± 0.00	7.24 ± 0.06	15.27 ± 0.12	309.33 ± 0.58	5.62 ± 0.24	0.06	0.16
	U.S	21.00 ± 0.00	7.43 ± 0.12	19.7 ± 0.00	304.33 ± 2.08	8.49 ± 0.47	0.86	0.23
	B.C	22.00 ± 0.00	7.67 ± 0.06	56.53 ± 0.12	229.33 ± 9.71	3.30 ± 0.97	0.05	2.11
APR	D.P	22.00 ± 0.00	7.40 ± 0.10	76.43 ± 0.29	311.11 ± 2.01	3.44 ± 0.67	0.05	2.10
	D.S	21.00 ± 0.00	7.63 ± 0.06	14.80 ± 0.00	151.00 ± 0.00	6.33 ± 0.21	0.32	0.38
	U.S	21.00 ± 0.00	6.91 ± 0.04	12.80 ± 0.00	20.22 ± 1.71	4.29 ± 0.79	1.03	1.08
	B.C	22.00 ± 0.00	7.08 ± 0.03	19.60 ± 0.00	38.22 ± 11.55	1.03 ± 0.19	0.16	2.72
MAY	D.P	21.00 ± 0.00	7.28 ± 0.02	13.80 ± 0.17	<10 ± 0.00	3.25 ± 0.17	0.20	2.34
	D.S	22.00 ± 0.00	7.13 ± 0.03	12.90 ± 0.00	309.11 ± 1.71	5.68 ± 0.30	1.72	0.82
	U.S	13.00 ± 0.00	7.65 ± 0.01	9.57 ± 0.01	112.89 ± 3.02	9.42 ± 0.15	2.40	2.17
	B.C	13.00 ± 0.00	7.37 ± 0.00	11.27 ± 0.31	300.00 ± 8.65	4.36 ± 0.16	5.98	12.38
JUN	D.P	12.00 ± 0.00	7.35 ± 0.01	8.92 ± 0.06	110.00 ± 3.06	4.54 ± 0.12	3.92	9.02
	D.S	14.00 ± 0.00	7.84 ± 0.01	14.37 ± 0.21	88.78 ± 2.41	7.74 ± 0.31	1.78	1.44
	U.S	15.00 ± 0.00	7.54 ± 0.01	13.27 ± 0.15	311.00 ± 1.00	5.76 ± 1.03	0.79	0.03
	B.C	16.00 ± 0.00	7.48 ± 0.01	19.33 ± 0.06	114.78 ± 11.65	2.56 ± 0.58	1.00	1.83
JUL	D.P	15.00 ± 0.00	7.70 ± 0.00	23.07 ± 0.38	290.67 ± 0.88	3.12 ± 0.62	0.89	0.75
	D.S	15.00 ± 0.00	7.87 ± 0.02	22.87 ± 0.12	312.44 ± 0.38	4.69 ± 0.23	1.35	0.09
	U.S	20.00 ± 0.00	7.12 ± 0.03	28.73 ± 0.06	105.89 ± 3.86	2.80 ± 0.57	0.06	0.12
	B.C	21.00 ± 0.00	6.85 ± 0.11	56.37 ± 0.35	310.11 ± 0.69	1.51 ± 1.09	0.00	0.00
AUG	D.P	19.00 ± 0.00	7.09 ± 0.4	65.53 ± 0.57	182.78 ± 2.27	1.59 ± 0.84	0.06	1.99
	D.S	20.00 ± 0.00	7.26 ± 0.02	20.77 ± 0.06	309.56 ± 2.14	3.98 ± 0.65	0.00	0.00
	U.S	20.00 ± 0.00	6.41 ± 0.05	10.67 ± 0.06	55.56 ± 0.51	3.73 ± 0.53	0.17	0.10
	B.C	22.00 ± 0.00	6.76 ± 0.02	20.73 ± 0.06	308.67 ± 0.88	1.51 ± 0.76	0.15	1.70
SEPT	D.P	20.00 ± 0.00	6.82 ± 0.04	19.27 ± 0.21	308.44 ± 1.26	2.38 ± 1.10	0.08	1.90
	D.S	20.00 ± 0.00	6.52 ± 0.02	11.50 ± 0.10	139.67 ± 1.73	3.92 ± 0.78	1.47	0.16
	U.S	24.00 ± 0.00	7.02 ± 0.01	17.07 ± 0.12	195.22 ± 3.98	3.32 ± 0.78	1.02	0.37
	B.C	22.00 ± 0.00	6.60 ± 0.06	30.53 ± 0.23	306.89 ± 1.84	3.23 ± 1.40	0.88	1.88
OCT	D.P	23.00 ± 0.00	6.75 ± 0.05	28.50 ± 0.00	109.89 ± 2.80	3.88 ± 0.75	0.82	1.57
	D.S	24.00 ± 0.00	6.91 ± 0.01	29.03 ± 0.06	148.00 ± 0.33	3.58 ± 0.98	1.30	0.42
	U.S	21.00 ± 0.00	6.86 ± 0.01	21.33 ± 0.76	241.78 ± 21.56	4.24 ± 0.98	0.97	0.64
NOV	B.C	22.00 ± 0.00	6.79 ± 0.01	39.13 ± 0.40	123.78 ± 6.91	3.56 ± 0.92	0.28	3.53
	D.P	23.00 ± 0.00	6.68 ± 0.03	48.53 ± 0.55	287.22 ± 14.25	3.26 ± 0.88	0.31	3.69
	D.S	23.00 ± 0.00	6.72 ± 0.05	14.10 ± 0.46	246.11 ± 14.84	3.87 ± 0.81	3.38	0.45
	U.S	22.00 ± 0.00	6.85 ± 0.01	12.20 ± 0.26	274.33 ± 4.41	3.65 ± 0.78	0.39	0.03
	B.C	25.00 ± 0.00	6.78 ± 0.03	36.13 ± 0.40	170.78 ± 3.79	3.29 ± 0.96	0.24	1.82
DEC	D.P	21.00 ± 0.00	6.69 ± 0.01	31.77 ± 0.23	153.89 ± 0.19	3.52 ± 0.77	0.77	1.37
	D.S	22.00 ± 0.00	6.64 ± 0.02	10.33 ± 0.41	205.33 ± 4.98	4.01 ± 0.79	1.95	0.35
	U.S	24.00 ± 0.00	7.04 ± 0.01	11.40 ± 0.26	299.22 ± 1.07	3.76 ± 0.67	0.58	0.23
JAN	B.C	24.00 ± 0.00	6.84 ± 0.01	12.67 ± 0.15	<10 ± 0.00	3.68 ± 0.94	0.02	4.70
	D.P	23.00 ± 0.00	6.87 ± 0.03	32.67 ± 0.81	303.67 ± 0.33	3.72 ± 0.95	0.02	4.69
	D.S	24.00 ± 0.00	6.92 ± 0.02	8.72 ± 0.04	150.11 ± 3.56	3.74 ± 0.66	1.22	0.12
	U.S	25.00 ± 0.00	7.41 ± 0.01	6.37 ± 0.02	308.89 ± 1.02	3.26 ± 0.39	0.22	0.03
FEB	B.C	25.00 ± 0.00	7.80 ± 0.01	40.37 ± 0.21	295.67 ± 4.73	1.82 ± 1.12	0.01	3.51
	D.P	25.00 ± 0.00	7.88 ± 0.01	44.07 ± 0.25	309.33 ± 0.58	2.81 ± 0.86	0.02	2.71
	D.S	27.00 ± 0.00	7.77 ± 0.01	5.94 ± 0.10	254.78 ± 5.39	4.01 ± 0.80	0.16	0.53

**Table 3:** Physicochemical parameters of wastewater effluent from New Germany wastewater treatment works and the receiving river from March 2012 to February 2013.

MONTH	Sampled Point	Parameter (Mean ± SD)						
		T (°C)	pH	T (ntu)	COD (mg/L)	BOD (mg/L)	NO_3_ (mg/L)	PO_4_ (mg/L)
	U.S	26.00 ± 0.00	7.52 ± 0.09	5.15 ± 0.05	313.89 ± 0.19	7.79 ± 0.83	1.06	0.05
MAR	B.C	26.00 ± 0.00	7.12 ± 0.21	6.65 ± 0.23	153.67 ± 11.46	2.20 ± 0.13	1.81	0.90
	D.P	26.00 ± 0.00	7.18 ± 0.12	5.71 ± 0.59	239.00 ± 10.00	3.12 ± 0.27	1.40	0.80
	D.S	26.00 ± 0.00	7.51 ± 0.09	7.32 ± 0.33	141.33 ± 11.06	4.97 ± 0.59	2.14	0.05
	U.S	18.00 ± 0.00	7.08 ± 0.02	8.23 ± 0.00	104.22 ± 2.83	8.49 ± 0.47	1.29	0.17
	B.C	20.00 ± 0.00	7.04 ± 0.04	1.52 ± 0.00	202.78 ± 9.10	3.30 ± 0.97	2.81	1.09
APR	D.P	19.00 ± 0.00	6.82 ± 0.01	1.42 ± 0.02	179.67 ± 1.20	3.44 ± 0.67	2.35	0.86
	D.S	20.00 ± 0.00	7.05 ± 0.04	17.00 ± 0.00	114.00 ± 1.73	6.33 ± 0.21	0.82	0.28
	U.S	16.00 ± 0.00	6.42 ± 0.05	3.18 ± 0.00	298.67 ± 0.33	11.04 ± 0.97	0.84	<0.025
	B.C	19.00 ± 0.00	6.91 ± 0.09	28.70 ± .00	312.22 ± 0.69	3.15 ± 0.25	<0.057	0.05
MAY	D.P	14.00 ± 0.00	7.02 ± 0.01	30.30 ± 0.00	246.33 ± 3.06	4.72 ± 0.16	<0.057	<0.025
	D.S	19.00 ± 0.00	7.10 ± 0.00	17.80 ± 0.00	311.89 ± 2.22	9.67 ± 0.55	0.51	<0.025
	U.S	16.00 ± 0.00	7.93 ± 0.01	9.02 ± 0.12	22.33 ± 3.79	10.80 ± 0.41	2.38	0.66
	B.C	18.00 ± 0.00	7.62 ± 0.01	9.63 ± 0.03	310.00 ± 1.73	4.19 ± 0.11	3.76	1.05
JUN	D.P	14.00 ± 0.00	7.55 ± 0.01	10.63 ± 0.51	137.67 ± 9.87	5.03 ± 0.07	1.99	0.88
	D.S	18.00 ± 0.00	7.83 ± 0.00	14.07 ± 0.12	73.33 ± 4.16	7.16 ± 1.57	2.15	0.33
	U.S	14.00 ± 0.00	6.30 ± 0.01	2.44 ± 0.01	309.67 ± 2.19	5.27 ± 0.41	1.03	0.03
	B.C	17.00 ± 0.00	6.53 ± 0.10	20.07 ± 0.12	193.67 ± 3.67	2.12 ± 0.17	0.47	0.03
JUL	D.P	15.00 ± 0.00	6.89 ± 0.01	20.73 ± 0.15	308.67 ± 0.58	3.95 ± 0.34	0.57	0.09
	D.S	17.00 ± 0.00	6.98 ± 0.02	16.10 ± 0.00	299.56 ± 4.62	9.42 ± 0.55	0.71	0.07
	U.S	15.00 ± 0.00	7.12 ± 0.03	40.40 ± 0.36	207.56 ± 1.07	3.27 ± 1.02	2.29	0.59
	B.C	17.00 ± 0.00	6.85 ± 0.11	19.73 ± 0.15	139.56 ± 1.02	3.62 ± 1.02	1.42	0.93
AUG	D.P	12.00 ± 0.00	7.26 ± 0.02	16.80 ± 0.17	309.00 ± 1.33	4.68 ± 0.80	1.96	1.47
	D.S	17.00 ± 0.00	7.09 ± 0.04	14.10 ± 0.10	311.78 ± 0.84	5.86 ± 1.57	0.80	1.89
	U.S	20.00 ± 0.00	6.48 ± 0.02	15.83 ± 0.15	310.33 ± 0.58	4.06 ± 0.91	2.65	0.54
	B.C	22.00 ± 0.00	6.75 ± 0.08	5.84 ± 0.01	98.22 ± 2.41	4.66 ± 0.84	8.22	0.58
**SEPT**	D.P	20.00 ± 0.00	6.37 ± 0.03	16.33 ± 0.06	310.44 ± 1.17	4.49 ± 1.08	0.23	0.14
	D.S	20.00 ± 0.00	6.59 ± 0.00	6.98 ± 0.02	189.89 ± 2.59	3.42 ± 0.47	0.72	0.17
	U.S	17.00 ± 0.00	6.97 ± 0.02	3.68 ± 0.01	311.89 ± 0.84	4.46 ± 0.67	2.32	0.05
	B.C	20.00 ± 0.00	6.91 ± 0.09	20.00 ± 0.10	35.67 ± 3.61	4.51 ± 0.84	1.23	1.12
**OCT**	D.P	19.00 ± 0.00	6.85 ± 0.14	6.48 ± 0.04	54.11 ± 3.15	4.42 ± 0.72	1.64	1.26
	D.S	20.00 ± 0.00	6.98 ± 0.03	5.10 ± 0.01	239.22 ± 4.81	4.79 ± 0.79	2.47	0.49
	U.S	17.00 ± 0.00	7.12 ± 0.01	8.11 ± 0.06	306.78 ± 2.46	4.31 ± 0.78	2.11	0.02
**NOV**	B.C	20.00 ± 0.00	6.82 ± 0.03	5.51 ± 0.08	69.11 ± 1.39	4.14 ± 0.61	0.50	3.22
	D.P	18.00 ± 0.00	7.14 ± 0.04	29.43 ± 0.06	108.56 ± 3.24	4.22 ± 0.71	0.33	3.99
	D.S	20.00 ± 0.00	7.16 ± 0.01	16.53 ± 0.23	257.56 ± 14.55	4.49 ± 0.81	1.27	1.70
	U.S	20.00 ± 0.00	6.47 ± 0.01	32.10 ± 0.10	24.33 ± 2.08	3.38 ± 1.46	<0.017	0.04
	B.C	22.00 ± 0.00	6.55 ± 0.28	4.41 ± 0.30	93.33 ± 5.93	2.87 ± 0.81	0.11	0.13
**DEC**	D.P	20.00 ± 0.00	6.45 ± 0.01	29.43 ± 0.06	300.44 ± 4.22	3.36 ± 0.92	<0.017	0.14
	D.S	22.00 ± 0.00	6.51 ± 0.04	28.10 ± 0.10	35.33 ± 3.84	4.17 ± 0.83	0.51	0.06
	U.S	22.00 ± 0.00	6.71 ± 0.01	10.80 ± 0.00	80.33 ± 5.13	3.92 ± 0.69	1.21	<0.08
**JAN**	B.C	23.00 ± 0.00	6.59 ± 0.02	9.43 ± 0.04	111.11 ± 0.84	3.43 ± 1.09	<0.017	0.01
	D.P	22.00 ± 0.00	6.61 ± 0.02	9.29 ± 0.04	240.78 ± 3.75	3.75 ± 0.79	<0.017	0.15
	D.S	23.00 ± 0.00	6.73 ± 0.00	10.60 ± 0.00	44.56 ± 3.42	4.50 ± 1.27	0.84	0.13
	U.S	21.00 ± 0.00	7.50 ± 0.05	8.83 ± 0.04	305.33 ± 0.58	3.04 ± 0.80	0.44	0.01
**FEB**	B.C	24.00 ± 0.00	7.72 ± 0.03	3.93 ± 0.01	158.89 ± 4.72	3.97 ± 1.10	<0.017	0.76
	D.P	23.00 ± 0.00	7.87 ± 0.02	4.02 ± 0.03	283.44 ± 3.79	4.08 ± 1.04	<0.017	1.16
	D.S	24.00 ± 0.00	8.08 ± 0.00	5.80 ± 0.02	272.89 ± 14.82	4.85 ± 0.85	0.39	0.64

Values are averages of three replicates ± standard deviation.

BOD values increased at the D.P during most of the study period but some level of decrease was recorded in September (17%) and October (9%). At the NWWTW, nitrate (NO_3_) values ranged from 0.00 mg/L at the B.C and D.S in August to 5.98 mg/L at the B.C in June, while phosphates (PO_4_) values ranged from 0 mg/L at the B.C and D.S in August to 12.38 mg/L. At the NGWTP, NO_3_ values ranged from <0.06 mg/L (D.P, May) to 8.22 mg/L (B.C, September) while recorded PO_4_ values ranged from <0.03 mg/L in May to 3.99 mg/L in November at the D.P (Table 3).

### 3.2. Enumeration of Presumptive Salmonella in Treated Wastewater Effluent and Receiving River

**Figure 1:**
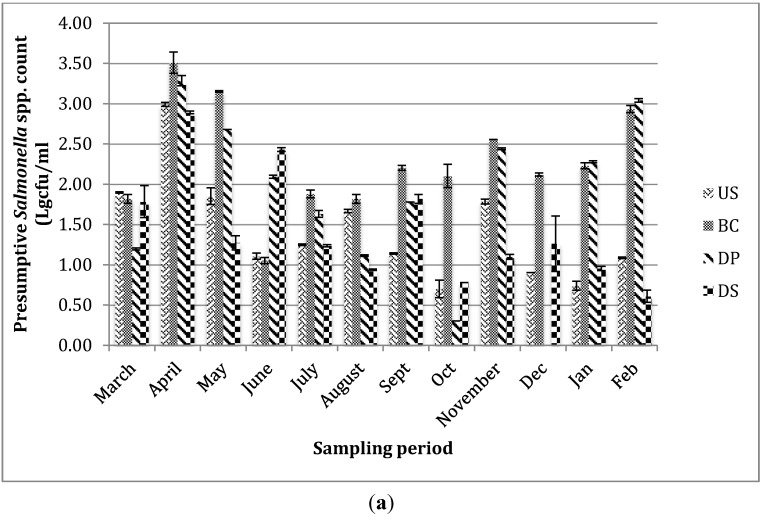

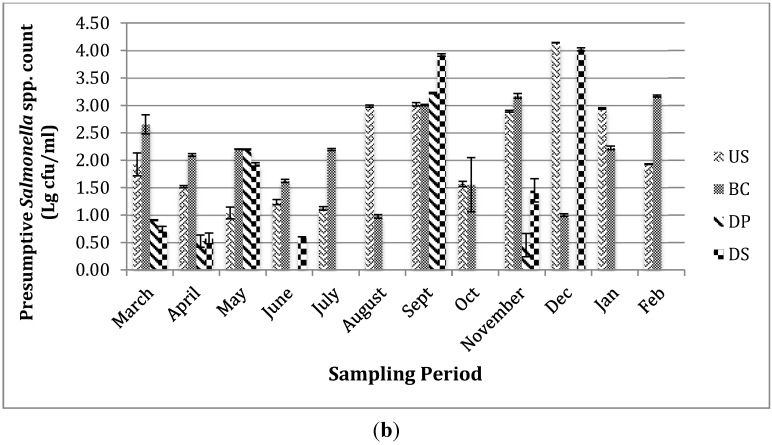
(**a**) Monthly variation of presumptive *Salmonella* spp. at (**a**) NWWTP and (**b**) NGWTP and their receiving surface waters during the study period.

The monthly variation of presumptive *Salmonella* spp. population in NWWTW and receiving Umgeni River are shown in Figure 1a. At U.S, counts for presumptive *Salmonella* spp. ranged from 0.70 log·cfu/mL in October to 2.99 log·cfu/mL in March, while at the BC point, counts ranged from 1.05 to 3.51 log·cfu/mL in June and May, respectively. At the D.P there was no presumptive *Salmonella* recorded in October while the highest count of 3.29 log·cfu/mL was recorded in April. At the D.S, the counts ranged from 0.61 log·cfu/mL in February to 2.98 log·cfu/mL in April. The monthly variation of presumptive *Salmonella* spp. population in NGWTP is given in Figure 1b. At the U.S, recorded values ranged from 1.04 log·cfu/mL in May to 3.02 log·cfu/mL in September. At the B.C point, the lowest count was recorded in August (0.98 log·cfu/mL) while the highest count was recorded in November (3.18 log·cfu/ml). No presumptive *Salmonella* was recorded at the D.P between June and August as well as December to February while the highest values was recorded in September (3.22 log·cfu/ml). At the D.S, the highest recorded value was obtained in the month of December (4.14 log·cfu/ml) while no *Salmonella* count was recorded in July, August, January and February.

### 3.3. Statistical Analysis

Table 4 and Table 5 show the correlation matrices of selected physicochemical parameters with microbial counts. At the NWWTW (Table 4), there was a strong positive correlation between Presumptive *Salmonella* counts and turbidity (r = 0.501) as well as with PO_4_ (r = 0.431). NO_3_ also strongly correlated positively with temperature (0.430) and BOD_5_ (r = 0.462). However, a strong inverse correlation was seen between Turbidity and BOD5 (r = −0.378) as well as NO_3_ (r = 0.408). Presumptive *Salmonella* counts strongly correlated negatively with both BOD_5_ (r = −0.325) and NO_3_ (r = −0.349). At the NGWTP (Table 5), PO_4_ correlated significantly (*p* > 0.05) with pH (r =0.302) but correlated strongly but negatively (*p* < 0.01) with temperature (r = −0.430).

**Table 4:** Correlation matrices of the physicochemical parameters at the NWWTW.

	Temp	pH	T	COD	BOD_5_	NO_3_	PO_4_	*Sal.* ^a^
Temp (°C)	1							
pH	0.140	1						
T (ntu)	0.065	−0.028	1					
COD (mg/L)	−0.031	−0.225	−0.205	1				
BOD_5_ (mg/L)	0.129	0.253	−0.378 **	0.036	1			
NO_3_ (mg/L)	0.430 **	0.011	−0.408 **	0.139	0.462 **	1		
PO_4_ (mg/L)	0.130	0.080	0.166	0.171	−0.169	0.118	1	
*Sal.* (cfu/mL)	−0.002	0.225	0.501 **	−0.078	−0.325 *	−0.349 *	0.431 **	1

**^a^**—*Salmonella* spp.; ** Correlation is significant at the 0.01 level (2-tailed); * Correlation is significant at the 0.05 level (2-tailed).

**Table 5:** Correlation matrices of physicochemical parameters at the NGWTP.

	Temp	pH	T	COD	BOD_5_	NO_3_	PO_4_	*Sal.* ^a^
Temp (°C)	1							
pH	0.094	1						
T (ntu)	−0.265	−0.148	1					
COD (mg/L)	0.262	−0.010	−0.024	1				
BOD_5_ (mg/L)	−0.419 **	0.264	−0.044	−0.195	1			
NO_3_ (mg/L)	−0.167	0.187	−0.240	0.046	0.151	1		
PO_4_ (mg/L)	−0.005	0.302 *	−0.158	0.213	−0.089	0.207	1	
*Sal.* (cfu/mL)	−0.002	−0.229	0.215	0.066	−0.246	−0.090	−0.161	1

**^a^**—*Salmonella* spp.; ** Correlation is significant at the 0.01 level (2-tailed); * Correlation is significant at the 0.05 level (2-tailed).

### 3.4. Distribution of Confirmed Salmonella spp. Recovered from Treated Wastewater and Receiving Surface Waters

After enrichment and confirmation, a total of 200 of the recovered isolates were confirmed as *Salmonella* spp. The NGWTP and receiving surface water has the highest prevalence (93.5%) of *Salmonella* spp.. while only 6.5% of the isolates were recovered in treated effluent before chlorination at the NWWTW. At the NGWTP, 53 (26.5%) of the isolates were recovered in effluent sample before chlorination. Also, 55 (27.5%) isolates were recovered at the discharge point of the NGWTP with additional 27% and 12.5% recovered upstream and downstream of the receiving river of the treated final effluent from the NGTWP, respectively.

### 3.5. Virulence Gene Distribution and Antibiotic Resistance Profiles of the Positively Identified Salmonella spp.

**Figure 2:**
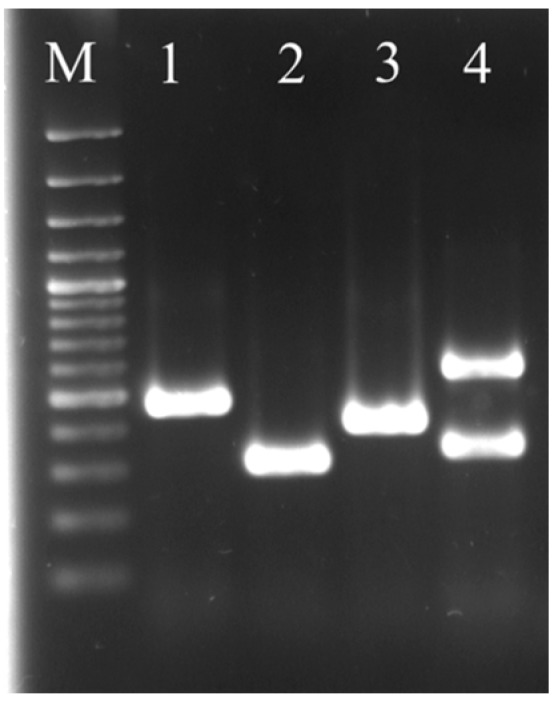
Representative agarose gel showing the *inv*A and virulence genes detected in the positively confirmed *Salmonella* spp. Lane M contains the 100 bp plus marker (Thermo Scientific), Lane 1 contains *inv*A gene (450bp), lane 2 contains *spi*C gene (309 bp), lane 3 contains *pip*D gene (400 bp), lane 4 contains the *mis*L (550 bp) and *orf*L (350 bp).

A representative gel of four virulence genes detected in *Salmonella* spp. is shown in Figure 2. Analysis of the virulence gene signatures in recovered *Salmonella* spp. revealed that 93% harbored the *spi*C gene, 84% harbored the *misL* gene, and 87.5% harbored the *orf*L gene while 87% harbored *pip*D gene. Antibiotic resistance profile of the 200 confirmed isolates is shown in Table 5.

The isolates were highly susceptible to β-lactams, chloramphenicol, tetracycline, norfloxacin and trimethoprim-sulfamethoxazole (99% to 100%). Resistance was observed against sulfamethoxazole (100%), nalidixic acid (27%) and streptomycin (14%). Resistance to third generation β-lactams was also observed in 0.5% of the isolates. Intermediate resistance was observed against streptomycin (74%), nalidixic acid (44%) and fosfomycin (8.5%). Though some of the isolates recovered were susceptible to nalidixic acid (54%), high resistance to third generation quinolones such as ciprofloxacin, norflorxacin and nitrofurantoin were not recorded (Table 6).

**Table 6:** Antibiotic resistance profile of *Salmonella* spp. recovered from wastewater treatment plants and receiving surface waters (n = 200).

Class	Antibiotics	Content	Resistant (%)	Intermediate (%)	Susceptible (%)
β-lactams	Cephalothin	30 μg	0 (0)	0 (0)	200 (100)
	Imipenem	10 μg	1 (0.5)	1 (0.5)	198 (99)
	Cefoxitin	30 μg	0 (0)	0 (0)	200 (100)
	Cefuroxime	30 μg	0 (0)	2 (1)	198 (99)
	Piperacillin	100 μg	1 (0.5)	3 (1.5)	196 (98)
	Ampicillin	10 μg	1 (0.5)	2 (1)	197 (98.5)
	Cefixime	5 μg	1 (0.5)	0 (0)	199 (99.5)
	Ceftazidime	30 μg	1 (0.5)	0 (0)	198 (99)
	Aztreonam	30 μg	0 (0)	0 (0)	200 (100)
Aminoglycosides	Gentamycin	(10 μg)	0 (0)	0 (0)	200 (100)
	Amikacin	30 μg	0 (0)	1 (0.5)	199 (99.5)
	Streptomycin	10 μg	28 (14)	148 (74)	24 (12)
Phenicols	Chloramphenicol	30 μg	0 (0)	0 (0)	200 (100)
Tetracyclines	Tetracycline	30 μg	0 (0)	0 (0)	200 (100)
Quinolones	Ciprofloxacin	5 μg	0 (0)	1 (0.5)	199 (99.5)
	Norflaoxacin	10 μg	0 (0)	0 (0)	200 (100)
	Nalidixic acid	30 μg	54 (27)	89 (44.5)	57 (28.5)
	Nitrofurantoin	30 μg	0 (0)	17 (8.5)	183 (91.5)
Sulfonamides	Trimethorprim/Sulfamethoxazole	1.25/23.75 μg	0 (0)	0 (0)	200 (100)
	Sulfamethoxazole	5 μg	200 (100)	0 (0)	0 (0)

## 4. Discussion and Conclusions

The impact of substandard quality effluent or untreated wastewater discharged into receiving water bodies can be detrimental to water availability and security. Wastewater management is the first barrier in a multi-barrier system to ensure safe drinking water, public health and environmental sustainability [32]. Physicochemical analysis of the wastewater gives an indication of the quality of effluents being discharge into the environment. The temperature regime varied depending on the season but was still within the acceptable limit of 25 °C [33] and did not pose any threat to the receiving watershed. The temperature is a very important parameter because of its effect on the chemical reaction and reaction rates, aquatic life and suitability of the water for beneficial uses. Increase in temperature can lead to the disruption of the ecosystem in the receiving watershed resulting in high mortality of aquatic life and encouraged growth of undesirable algae and wastewater fungi [34]. At both plants, the pH ranged between 6.41–8.08 and 6.37–7.88. The neutral to alkaline pH values recorded in this study are similar to previous reports [7]. Very low or high pH is known to be toxic to aquatic life and alters the solubility of chemicals in water [35]. The pH of most natural waters is in the range of 4−9 and the target limit set by the South African Department of Water Affairs is between 5.5 and 9.5 [33]. The pH of water can provide important information about many chemical and biological processes and provides indirect correlations to a number of different impairments in the wastewater treatment processes [36]. Changes in pH can also be indicative of industrial pollution, photosynthesis or the decomposition of organic matter by microorganisms [37]. Hence, the pH values recorded in this study fell within the acceptable range indicating that discharge of the treated wastewater may have no negative impact on the river water with respect to pH.

The turbidity of the water samples in this study ranged between 1.42 NTU to 76.43 NTU and varied seasonally (Table 2 and Table 3). Higher turbidity values recorded in some months at the D.P could be the result of storm runoff and inadequate treatment leading to the high variation in turbidity. This high variation has been reported in previous studies in the Eastern Cape province of South Africa [7]. The turbidity values recorded in this study was higher than the <5 NTU set by the World Health Organization (WHO) for effluent discharge into the environment [7]. Turbidity is caused by small particles that may be organic or inorganic and can provide food and shelter for pathogens providing a possible explanation of its strong positive correlation with *Salmonella* count at the NWWTW (Table 4). If not removed, turbidity can promote the regrowth of pathogens in the final effluent of receiving water body into which the effluent is discharged [38]. Turbidity also limits the bactericidal effect of chlorine in the wastewater during disinfection [35] and may react with organic compounds in the water to form micro-contaminants such as trihalomethane (THM). THMs are carcinogenic and render the water unfit for recreational activities to consumers who may depend on the receiving water shed for such [9,39].

Although the BOD_5_ values recorded was stable across each sampling point in each month, the values varied significantly in the course of the study ranging from 1.03 mg/L to 11.04 mg/L. There is no South African guideline for BOD_5_ levels in the final effluent of wastewater; however, the EU recommends a discharge limit of 3 to 6 mg/L for aquatic ecosystems [6]. Discharge of effluent high in BOD into water bodies would result in rapid depletion of dissolved oxygen leading to anoxic conditions, and consequent disruption of balance in the aquatic ecosystem [6]. On most occasions the recorded BOD_5_ values at the D.P were within the recommended EU limit. The COD of the water samples varied remarkably throughout the study period. High COD values were recorded at U.S while, the average recorded values (212 mg/L) at the D.P greatly exceeded the South African limit of 30 mg/L [33]. High levels of COD observed U.S could be attributed to runoff, agricultural activities and anthropogenic activities. Igbinosa and Okoh [7] reported a similar observation and attributed the increased COD to addition of organic and inorganic substances from the environment as well as organic contaminants entering the system from municipal sewage treatment plants or non-point sources of pollution. Higher average COD values varying from 512 to 698.11 mg/L were reported in a study on river quality in India and was attributed to the presence of inorganic chemicals in the wastewater of a nearby chemical industry [40].

Tertiary treatment of final sewage effluent with chlorine at the wastewater treatment plants (WWTPs) under investigation reduced the number of viable presumptive *Salmonella* spp. at the discharge point during the sampling period but failed to totally eliminate them (Figure 1). Presumptive *Salmonella* spp. were also recovered downstream of the receiving rivers indicating the negative impact of the wastewater treatment plants on the microbial quality of the river. This indicates that treated wastewater effluents discharged from these treatment plants are a source of contamination of receiving surface waters with this potential pathogen. Upstream of the river at the NGWTP is an informal settlement with poor sanitation and inadequate sewage disposal system, which contaminates the river with human and animal wastes while the bank of the Umgeni River downstream, is littered with feces. Storm runoff from this informal settlement and riverbanks explains the high count of presumptive *Salmonella* spp. observed upstream. In contrast to findings from this study, Olaniran, Naidoo and Pillay [24] reported lower counts of *Salmonella* spp. from treated wastewater of same plants under investigation, which may be due to the short duration of the study or an improvement in the operational conditions of the treatment plants. Elsewhere, Momba, Osode and Sibewu [6] reported recovery of pathogenic microorganisms including *Salmonella* spp. in the final effluents of four WWTPs in the Eastern Cape province of South Africa and concluded that WWTPs serve as a point source of microbial pollution of water bodies. Recent reports have also suggested that WWTPs plants in South Africa are either dysfunctional or non-functional [41], inefficient in removing microbial pathogens from wastewater and produce wastewater effluent of unacceptable standard that does not meet discharge guidelines set by the department of water affairs, South Africa [42,43,44,45]. The issue of treatment efficiency is of major importance if the reclaimed water is intended for recreational or potable reuse or is to be discharged into water bodies because disposal of inadequately treated wastewater into surface water recipient is one of the major sources of pathogens in the environment [42,46]. Though *Salmonella* is isolated from water in lower numbers than indicator bacteria such as fecal coliform, fecal streptococci and enterococci; counts in the range of 15−1000 cfu/mL may pose public health risks [47]. Thus, the presence of this organism in the final effluent of wastewater and receiving surface water is a serious cause for concern especially where the contaminated water is depended on for irrigation and rural socio-economic activities.

In this study, 200 *Salmonella* spp. were recovered and found to contain the *inv*A gene (Figure 2), which led to their confirmation as *Salmonella* spp. in agreement with previous studies [48,49]. The *inv*A gene is conserved in all *Salmonella* spp. and encodes for a protein in the inner and outer membrane which is essential for virulence and is thought to trigger the internalization required for invasion into deeper tissues [29,30]. At the NWWTW, confirmed *Salmonella* spp. were only recovered at the B.C point (6.5%) but not at the D.P indicating the plant was efficient at removing *Salmonella* spp. from the wastewater during the sampling period. This is also corroborated by the fact that the NWWTW has a high Greendrop status rating [50]. The Greendrop is an index developed by the Department of Water Affairs in South Africa to rate wastewater treatment plants in the country based on the quality of their final effluent. In the Eastern Cape province of South Africa, Momba, Osode and Sibewu [6] observed the presence of *Salmonella* spp. in 50% of final wastewater effluent and 35% in the receiving river samples. Poor sanitation, lack of access to proper sewage disposal systems, malnutrition and poverty have been described as some of the leading factors contributing to the high prevalence of salmonellosis and other diarrheal diseases in developing countries [51,52,53].

Amplification of virulence genes (Figure 2) revealed that the isolates contained one or more virulence genes present in the *Salmonella* pathogenicity island (SPI). This can pose serious health threats to consumers who depend on the river water for daily and recreational activities. Pathogenicity islands are found on genomes of pathogenic bacteria but are absent in non-pathogenic strains of the same or related species [54]. The presence of all four virulence genes were reported to be present in 87.2% of *Salmonella* isolated from patients with systemic infection [55] while 12.8% of *Salmonella* spp. isolated from stool samples lacked the *mis*L and *orf*L gene. The *spi*C gene is found in the SPI-2 and is essential for systemic pathogenesis because it encodes a type III secretion system (T3SS) that is activated after invasion [56,57]. The T3SS system is used by the pathogen to deliver virulence factors to the host cell and interfere with or subvert normal host cell signaling pathways [58]. The *mis*L gene located on SPI-3 encodes an autotransporter protein involved in intestinal colonization and essential for survival in macrophages [55,59]. The SPI-4 is a 25 kb pathogenicity island containing the *orf*L gene thought to encode a type 1 secretion system (an autotransporter protein) that mediate the secretion of toxins and is necessary for macrophage survival [55,59], while the *pip*D gene encodes effector proteins for the T3SS transport protein [29,30] and is mainly associated with enteropathogenesis [58]. The presence of these virulence genes in *Salmonella* spp. isolated from treated wastewater effluent and receiving surface water indicate the capabilities of these isolates in causing infections in susceptible hosts. Recently, there was report of an outbreak of acute gastroenteritis in KwaZulu-Natal, which was linked to food, contaminated with *Salmonella enterica serovar Enteritidis* resulting in the hospitalization of 216 people [60]. The report suggested a point source outbreak with a possibility of continued transmission. The true burden of *Salmonella* disease in Africa is unclear thus a comprehensive epidemiological study is needed to elucidate it. The *Salmonella* serotypes that most commonly cause invasive non-typhoidal *Salmonella* in Africa are *S. typhimurium* and *S. enteritidis*, which are usually associated with a broad host range and with enteric diseases [11].

Resistance against sulfamethoxazole (100%), nalidixic acid (27%) and streptomycin (14%) was observed among these isolates. Intermediate resistance was observed against streptomycin (74%), nalidixic acid (44%) and fosfomycin (8.5%). Resistance to nalidixic acid suggests possible resistance to third generation quinolones CLSI [31]. However, this was not observed in this study, as isolates resistant to nalidixic acid were also susceptible to the third generation quinolones tested. Previous studies have suggested that quinolones should not be used in the treatment of invasive Salmonellosis due to strains with decreased sensitivity to fluoroquinolones and possible risk of treatment failure [29,61]. The results obtained in this study further emphasize the need for prudent use of fluoroquinolones and other commonly used antibiotics to prevent the emergence of resistant phenotypes [62]. Consistent with this study, *Salmonella* spp. were reported to be highly sensitive to third generation β-lactams [29,59] but resistant to sulfamethoxazole, nalidixic acid and streptomycin. Data from the National Antimicrobial Resistance Monitoring Systems (NARMS) in the US from 1996 to 2004 showed increase in resistance of clinical isolates of *Salmonella* against antibiotics [63]. The upsurge in multidrug resistant strains of *Salmonella* over the past decade is threatening successful treatment of diseases caused by this organism especially in developing countries where disease burden is high [64].

In conclusion, this study revealed that treated effluents from WWTPs investigated are reservoirs of antibiotic resistant and virulent *Salmonella* spp. The isolates were susceptible to most third generation β lactams tested whilst exhibiting resistance to other antibiotics. The presence of virulence genes is indicative of their capabilities to cause infection in susceptible hosts. Though certain physicochemical parameters were within the stipulated guidelines, the overall quality of the treated effluent is still low. Thus, in order to protect the valuable surface water resources, urgent intervention is required by the regulatory authorities and workers in these treatment plants to optimize treatment efficiency. Constant surveillance of the treatment processes and final effluent, infrastructural upgrade of the wastewater treatment works and provision of adequate sanitation and sewage disposal systems to rural communities on the banks of rivers is recommended.

## References

[1] Pitman W. (2011). Overview of water resource assessment in South Africa: Current state and future challenges. Water SA.

[2] Adewumi J.R., Ilemobade A.A., van Zyl J.E. (2010). Treated wastewater reuse in South Africa: Overview, potential and challenges. Resour. Conserv. Recycl..

[3] Ngwa G.A., Schop R., Weir S., León-Velarde C.G., Odumeru J.A. (2013). Detection and enumeration of *E. Coli* O157:H7 in water samples by culture and molecular methods. J. Microbiol. Methods.

[4] Agrafioti E., Diamadopoulos E. (2012). A strategic plan for reuse of treated municipal wastewater for crop irrigation on the island of crete. Agric. Water Manag..

[5] Levantesi C., Bonadonna L., Briancesco R., Grohmann E., Toze S., Tandoi V. (2012). *Salmonella* in surface and drinking water: Occurrence and water-mediated transmission. Food Res. Int..

[6] Momba M.N.B., Osode A.N., Sibewu M. (2006). The impact of inadequate wastewater treatment on the receiving water bodies—Case study: Buffalo City and Nkokonbe Municipalities of the Eastern Cape Province. Water SA.

[7] Igbinosa E.O., Okoh A.I. (2009). Impact of discharge wastewater effluents on the physico-chemical qualities of a receiving watershed in a typical rural community. Int. J. Environ. Sci. Technol..

[8] Petala M., Kokokiris L., Samaras P., Papadopoulos A., Zouboulis A. (2009). Toxicological and ecotoxic impact of secondary and tertiary treated sewage effluents. Water Res..

[9] Ratola N., Cincinelli A., Alves A., Katsoyiannis A. (2012). Occurrence of organic microcontaminants in the wastewater treatment process. A mini review. J. Hazard. Mater..

[10] George I., Crop P., Servais P. (2002). Fecal coliform removal in wastewater treatment plants studied by plate counts and enzymatic methods. Water Res..

[11] Bertuzzo E., Azaele S., Maritan A., Gatto M., Rodriguez-Iturbe I., Rinaldo A. (2008). On the space-time evolution of a cholera epidemic. Water Resour. Res..

[12] Soyer Y., Orsi R.H., Rodriguez-Rivera L.D., Sun Q., Wiedmann M. (2009). Genome wide evolutionary analyses reveal serotype specific patterns of positive selection in selected *Salmonella* serotypes. BMC Evol. Biol..

[13] Fookes M., Schroeder G.N., Langridge G.C., Blondel C.J., Mammina C., Connor T.R., Seth-Smith H., Vernikos G.S., Robinson K.S., Sanders M. (2011). *Salmonella bongori* provides insights into the evolution of the *Salmonellae*. PLoS Pathog..

[14] Majowicz S.E., Musto J., Scallan E., Angulo F.J., Kirk M., O’Brien S.J., Jones T.F., Fazil A., Hoekstra R.M., International Collaboration on Enteric Disease “Burden of Illness” Studies (2010). The global burden of nontyphoidal *Salmonella* gastroenteritis. Clin. Infect. Dis..

[15] Kotloff K.L., Blackwelder W.C., Nasrin D., Nataro J.P., Farag T.H., van Eijk A., Adegbola R.A., Alonso P.L., Breiman R.F., Golam Faruque A.S. (2012). The Global Enteric Multicenter Study (GEMS) of diarrheal disease in infants and young children in developing countries: Epidemiologic and clinical methods of the case/control study. Clin. Infect. Dis..

[16] O’Brien S.J. (2013). The “decline and fall” of nontyphoidal *Salmonella* in the United Kingdom. Clin. Infect. Dis..

[17] Oluyege J.O., Dada A.C., Odeyemi A.T. (2009). Incidence of multiple antibiotic resistant gram-negative bacteria isolated from surface and ground water sources in south western region of Nigeria. Water Sci. Technol..

[18] Duffy L.L., Dykes G.A., Fegan N. (2012). A review of the ecology, colonization and genetic characterization of *Salmonella enterica* serovar Sofia, a prolific but avirulent poultry serovar in Australia. Food Res. Int..

[19] Oteo J., Lázaro E., de Abajo F.J., Baquero F., Campos J., Spanish Members of EARSS (2005). Antimicrobial-resistant invasive *Escherichia coli*, Spain. Emerg. Infect. Dis..

[20] Hendricks R., Pool E.J. (2012). The effectiveness of sewage treatment processes to remove faecal pathogens and antibiotic residues. J. Environ. Sci. Health Pt. A Toxic..

[21] Wani D., Pandit A.K., Kamili A.N. (2013). Microbial assessment and effect of seasonal change on the removal efficiency of FAB based sewage treatment plant. J. Environ. Eng. Ecol. Sci..

[22] Godinho V., Nascimento F., Silva S., von Sperling M. (2010). Characterisation of pathogenic bacteria in a UASB-polishing pond system using molecular techniques. Water Sci. Technol..

[23] Levantesi C., la Mantia R., Masciopinto C., Böckelmann U., Ayuso-Gabella M.N., Salgot M., Tandoi V., van Houtte E., Wintgens T., Grohmann E. (2010). Quantification of pathogenic microorganisms and microbial indicators in three wastewater reclamation and managed aquifer recharge facilities in Europe. Sci. Total Environ..

[24] Olaniran A.O., Naidoo S., Pillay B. (2012). Surveillance of invasive bacterial pathogens and human enteric viruses in wastewater final effluents and receiving water bodies—A case study from Durban, South Africa. CLEAN—Soil Air Water.

[25] Brichta-Harhay D.M., Arthur T.M., Bosilevac J.M., Guerini M.N., Kalchayanand N., Koohmaraie M. (2007). Enumeration of *Salmonella* and *Escherichia coli* O157:H7 in ground beef, cattle carcass, hide and faecal samples using direct plating methods. J. Appl. Microbiol..

[26] Espigares E., Bueno A., Espigares M., Gálvez R. (2006). Isolation of *Salmonella* serotypes in wastewater and effluent: Effect of treatment and potential risk. Int. J. Hyg. Environ. Health.

[27] Akinbowale O.L., Peng H., Grant P., Barton M.D. (2007). Antibiotic and heavy metal resistance in motile aeromonads and pseudomonads from rainbow trout (Oncorhynchus mykiss) farms in Australia. Int. J. Antimicrob. Agents.

[28] Gassama-Sow A., Wane A.A., Canu N.A., Uzzau S., Kane A.A., Rubino S. (2006). Characterization of virulence factors in the newly described *Salmonella enterica* serotype keurmassar emerging in Senegal (sub-Saharan Africa). Epidemiol. Infect..

[29] Lee Y.J., Kim H.J., Park C.K., Kim K.S., Bae D.H., Kang M.S., Cho J.K., Kim A.R., Kim J.W., Kim B.H. (2007). Characterization of *Salmonella* spp. Isolated from an integrated broiler chicken operation in Korea. J. Vet. Med. Sci..

[30] Dione M.M., Ikumapayi U., Saha D., Mohammed N.I., Adegbola R.A., Geerts S., Ieven M., Antonio M. (2011). Antimicrobial resistance and virulence genes of non-typhoidal *Salmonella* isolates in the gambia and senegal. J. Infect. Dev. Ctries.

[31] (2007). Performance Standards for Antimicrobial Susceptibility Testing; Seventeenth Informational Supplement. CLSI M100-S17. Clinical and Laboratory Standard Institute document. http://microbiolab-bg.com/wp-content/uploads/2015/05/CLSI.pdf.

[32] (2010). Wastewater Risk Abatement Plan: A Guideline to Plan and Manage Towards Safe and Compliant Wastewater Collection and Treatment in South Africa.

[33] (1984). General and special standards. Government Gazette.

[34] Ntengwe F.W. (2005). The cost benefit and efficiency of waste water treatment using domestic ponds—The ultimate solution in southern Africa. Phys. Chem. Earth Pt. A/B/C.

[35] Odjadjare E.O., Okoh A. (2010). Physicochemical quality of an urban municipal wastewater effluent and its impact on the receiving environment. Environ. Monit. Assess..

[36] Annalakshmi G., Amsath A. (2012). An assessment of water quality of river cauvery and its tributaries arasalar with reference to physico-chemical parameters at Tanjore DT, Tamilnadu, India. Int. J. Appl. Biol. Pharm. Technol..

[37] Oyhakilome G.I., Aiyesanmi A.F., Coolborn A.F. (2012). Water quality assessment of the Owena multi-purpose Dam, Ondo State, southwestern Nigeria. J. Environ. Prot..

[38] Altaher H., Alghamdi A. (2011). Enhancement of quality of secondary industrial wastewater effluent by coagulation process: A case study. J. Environ. Prot..

[39] Baršienė J., Andreikėnaitė L., Vosylienė M.Z., Milukaitė A. (2009). Genotoxicity and immunotoxicity of wastewater effluents discharged from vilnius wastewater treatment plant. Acta Zool. Litu..

[40] Singh N.S., Srivastava G., Bhatt A. (2012). Physicochemical determination of pollutants in wastewater in Dheradun. Curr. World Environ..

[41] Bateman C. (2010). Second report slams crippling neglect of water/sanitation systems. South Afr. Med. J..

[42] Odjadjare E.E., Igbinosa E.O., Mordi R., Igere B., Igeleke C.L., Okoh A.I. (2012). Prevalence of multiple antibiotics resistant [MAR] pseudomonas species in the final effluents of three municipal wastewater treatment facilities in South Africa. Int. J. Environ. Res. Public Health.

[43] Igbinosa E.O., Obi L.C., Okoh A.I. (2009). Occurrence of potentially pathogenic vibrios in final effluents of a wastewater treatment facility in a rural community of the Eastern Cape Province of South Africa. Res. Microbiol..

[44] Samie A., Obi C.L., Igumbor J.O., Momba M.N.B. (2009). Focus on 14 sewage treatment plants in the Mpumalanga province, South Africa in order to gauge the efficiency of wastewater treatment. Afr. J. Biotechnol..

[45] Dungeni M., Momba M.N.B. (2010). The Efficiency of Waste Water Treatment Systems in Rural and Urban Areas in the Removal of Cryptosporidium and Giardia Species. Water SA.

[46] Naidoo S., Olaniran A.O. (2014). Treated wastewater effluent as a source of microbial pollution of surface water resources. Int. J. Environ. Res. Public Health.

[47] Girones R., Ferrús M.A., Alonso J.L., Rodriguez-Manzano J., Calgua B., de Abreu Corrêa A., Hundesa A., Carratala A., Bofill-Mas S. (2010). Molecular detection of pathogens in water—The pros and cons of molecular techniques. Water Res..

[48] Deekshit V.K., Kumar B.K., Rai P., Rohit A., Karunasagar I. (2013). Simultaneous detection of *Salmonella* pathogenicity island 2 and its antibiotic resistance genes from seafood. J. Microbiol. Methods.

[49] Turki Y., Ouzari H., Mehri I., Ben Aissa R., Hassen A. (2012). Biofilm formation, virulence gene and multi-drug resistance in *Salmonella* kentucky isolated in Tunisia. Food Res. Int..

[50] (2009). Green Drop Report South Africa Wastewater Quality Performance.

[51] Woldemicael G. (2011). Diarrhoeal morbidity among young children in Eritrea: Environmental and socioeconomic determinants. J. Health Popul. Nutr..

[52] Wake M., Tolessa C. (2012). Reducing diarrhoeal diseases: Lessons on sanitation from Ethiopia and Haiti. Int. Nurs. Rev..

[53] Ijaz M.K., Rubino J.R. (2012). Impact of infectious diseases on cognitive development in childhood and beyond: Potential mitigational role of hygiene. Open Infect. Dis. J..

[54] Dobrindt U., Reidl J. (2000). Pathogenicity islands and phage conversion: Evolutionary aspects of bacterial pathogenesis. Int. J. Med. Microbiol..

[55] Sánchez-Jiménez M.M., Cardona-Castro N.M., Canu N., Uzzau S., Rubino S. (2010). Distribution of pathogenicity islands among colombian isolates of *Salmonella*. J. Infect. Dev. Ctries.

[56] Hensel M. (2004). Evolution of pathogenicity islands of *Salmonella enterica*. Int. J. Med. Microbiol..

[57] Miki T., Okada N., Shimada Y., Danbara H. (2004). Characterization of *Salmonella* pathogenicity island 1 type III secretion-dependent hemolytic activity in *Salmonella enterica* serovar typhimurium. Microb. Pathog..

[58] Marcus S.L., Brumell J.H., Pfeifer C.G., Finlay B.B. (2000). *Salmonella* pathogenicity islands: Big virulence in small packages. Microb. Infect..

[59] Xia X., Zhao S., Smith A., McEvoy J., Meng J., Bhagwat A.A. (2009). Characterization of *Salmonella* isolates from retail foods based on serotyping, pulse field gel electrophoresis, antibiotic resistance and other phenotypic properties. Int. J. Food Microbiol..

[60] Niehaus A.J., Apalata T., Coovadia Y.M., Smith A.M., Moodley P. (2011). An outbreak of foodborne *Salmonellosis* in rural Kwazulu-Natal, South Africa. Foodborne Pathog. Dis..

[61] Tajbakhsh M., Hendriksen R., Nochi Z., Zali M., Aarestrup F., Garcia-Migura L. (2012). Antimicrobial resistance in *Salmonella* spp. Recovered from patients admitted to six different hospitals in Tehran, Iran from 2007 to 2008. Folia Microbiol..

[62] Hur J., Jawale C., Lee J.H. (2012). Antimicrobial resistance of *Salmonella* isolated from food animals: A review. Food Res. Int..

[63] (2007). Bacterial Foodborne and Diarrheal Disease National Case Surveillance.

[64] Wellington E.M.H., Boxall A.B.A., Cross P., Feil E.J., Gaze W.H., Hawkey P.M., Johnson-Rollings A.S., Jones D.L., Lee N.M., Otten W. (2013). The role of the natural environment in the emergence of antibiotic resistance in gram-negative bacteria. Lancet Infect. Dis..

